# The perceived impact of the Covid-19 pandemic on medical student education and training – an international survey

**DOI:** 10.1186/s12909-021-02983-3

**Published:** 2021-11-09

**Authors:** TMS Collaborative, Kasun Wanigasooriya, William Beedham, Ryan Laloo, Rama Santhosh Karri, Adnan Darr, Georgia R. Layton, Peter Logan, Yanyu Tan, Devender Mittapalli, Tapan Patel, Vivaswan Dutt Mishra, Osama Odeh, Swathi Prakash, Salma Elnoamany, Sri Ramya Peddinti, Elorm Adzoa Daketsey, Shardool Gadgil, Ahmad Elmabri Mohammad Bouhuwaish, Ahmad Ozair, Sanchit Bansal, Muhammed Elhadi, Aditya Amit Godbole, Ariana Axiaq, Faateh Ahmad Rauf, Ashna Ashpak

**Affiliations:** 1 The Master Surgeon Trust, Birmingham, Worcestershire UK; 2 College of Medical and Dental Science, University of Birmingham, B15 2TT, Birmingham, UK; 3 Leeds Vascular Institute, Leeds General Infirmary, Leeds, UK; 4 The Royal Wolverhampton NHS Trust, Wolverhampton, UK; 5 Department of Cardiac Surgery, University Hospitals of Leicester NHS Trust, Leicester, UK; 6 Walsall Healthcare NHS Trust, Walsall, UK; 7 North East Deanery, Newcastle upon Tyne, UK; 8 University Hospitals Plymouth NHS Trust, Plymouth, UK; 9 Baroda Medical College, Vadodara, India; 10 Motilal Nehru Medical College, Allahabad, India; 11 University of Jordan, Amman, Jordan; 12 HCG Cancer Centre, Bangalore, India; 13 Faculty of Medicine, Menoufia University, Menoufia, Egypt; 14 Indira Gandhi Medical College and Research Institute, Puducherry, India; 15 Ysbyty Gwynedd, Betsi Cadwaladr University Health Board, Gwynedd, Bangor, North Wales UK; 16 Lokmanya Tilak municipal medical college, Mumbai, India; 17 Faculty of Medicine Tobruk University, Tobruk, Libya; 18 Faculty of Medicine, King George’s Medical University, Lucknow, Uttar Pradesh India; 19 Vardhman Mahavir Medical College and Safdarjung Hospital, New Delhi, India; 20 Faculty of Medicine, University of Tripoli, Tripoli, Libya; 21 Bharati Vidyapeeth (Deemed to be University) Medical College, Dhankawadi, Pune, India; 22 School of Medicine, Faculty of Life Sciences, Queen’s University Belfast, Belfast, UK; 23 Combined Military Hospital Lahore Medical College, Lahore, Pakistan; 24 School of Medicine, University of Central Lancashire, Preston, Lancashire UK

**Keywords:** Medical students, Undergraduate, Medical education, Covid-19, Cross-sectional survey

## Abstract

**Background:**

The Covid-19 pandemic led to significant changes and disruptions to medical education worldwide. We evaluated medical student perceived views on training, their experiences and changes to teaching methods during the pandemic.

**Methods:**

An online survey of medical students was conducted in the Autumn of 2020. An international network of collaborators facilitated participant recruitment. Students were surveyed on their perceived overall impact of Covid-19 on their training and several exposure variables. Univariate analyses and adjusted multivariable analysis were performed to determine strengths in associations.

**Results:**

A total of 1604 eligible participants from 45 countries took part in this survey and 56.3% (*n* = 860) of these were female. The median age was 21 (Inter Quartile Range:21–23). Nearly half (49.6%, *n* = 796) of medical students were in their clinical years. The majority (*n* = 1356, 84.5%) were residents of a low or middle income country. A total of 1305 (81.4%) participants reported that the Covid-19 pandemic had an overall negative impact on their training. On adjusted analysis, being 21 or younger, females, those reporting a decline in conventional lectures and ward based teaching were more likely to report an overall negative impact on their training (*p ≤* 0.001). However, an increase in clinical responsibilities was associated with lower odds of participants reporting a negative impact on training (*p* < 0.001). The participant’s resident nation economy and stage of training were associated with some of the participant training experiences surveyed (*p* < 0.05).

**Conclusion:**

An international cohort of medical students reported an overall significant negative impact of the Covid-19 pandemic on their undergraduate training. The efficacy of novel virtual methods of teaching to supplement traditional teaching methods warrants further research.

**Supplementary Information:**

The online version contains supplementary material available at 10.1186/s12909-021-02983-3.

## Background

In 2020, the Coronavirus Disease 2019 (Covid-19) pandemic led to the disruption of medical education and training worldwide [1]. Medical students experienced significant interruptions to their training given lengthy national lockdowns and strict social distancing measures [2]. Preliminary research has demonstrated that medical students faced exam cancellations and postponed placements [3]. Many were delayed from progressing to the next stage of training including applying for postgraduate training or residency programmes [4]. The pandemic also likely had an impact on medical student physical and psychological wellbeing [5]. Students were exposed to the virus and may have contracted Covid-19 either during their placements or at populous academic institutions [6]. Others reported feeling disheartened and emotionally detached during lockdown [7]. Many were also concerned about returning to placement due to low motivation and perceived risk to patients [8].

The increase in demand on acute healthcare services to provide care for Covid-19 patients likely resulted in medical student teaching taking a lower priority. Therefore, higher academic institutions delivering medical student curricula were forced to adapt their teaching methods in response to the pandemic over the past year but the efficacy of these new measures remain untested [9]. Several novel virtual teaching methods such as online lectures, tutorials, webinars and courses have replaced traditional face to face teaching [10, 11]. The extent to which these novel methods are being used to train medical students worldwide is yet unknown. Furthermore, students have expressed concerns regarding the efficacy of such methods in delivering holistic undergraduate medical training [12]. A recent survey of medical students from the United States (US) found that 43.3% felt unprepared for their clerkships and 56.7% felt unprepared for their US Medical Licensing Exam following virtual teaching during the Covid-19 pandemic [13]. Therefore, we proceeded to survey undergraduate medical students worldwide on the overall perceived impact of the Covid-19 pandemic on their medical student training; changes to teaching methods, clinical responsibilities and other training experiences during the pandemic.

## Methods

### Survey design and setting

This electronic cross-sectional survey was designed and conducted by The Master Surgeon Trust Collaborative (The Master Surgeon Trust, United Kingdom [UK], HMRC small medical education charity reference: EW03332). Research ethics committee approval was not required for this survey and this was confirmed using the UK, Health Research Authority “Is my study research?” online decision tool (http://www.hra-decisiontools.org.uk/research; Supplementary Document 1). Informed consent was obtained from all participants and recorded electronically at the start of the survey. The survey data was processed confidentially, anonymously and in compliance with the General Data Protection Regulations of the European Union. The survey was administered using the SurveyMonkey (San Mateo, California, USA) online survey administration and management platform between the 04th of August 2020 and 17th November 2020. The survey was conducted in the English language and took approximately 10 min to complete. The questionnaire can be found in the supplementary documents (Supplementary Document 2). A survey based, qualitative methodology was chosen due to cost-effectiveness and administrative convenience. Others have used similar methods to evaluate the Covid-19 pandemic impact on various groups [3, 14].

### Survey participation

Medical students worldwide, aged 18 or over and enrolled in a nationally or internationally accredited medical school were eligible to take part in this survey. Eligible students were in pre-clinical or clinical years in a medical course leading to a qualification enabling them to practice medicine. Students enrolled in “pre-med” courses were not eligible to take part in this survey. The study was promoted via email and through social media. An international team of volunteer collaborators distributed the survey to eligible participants within their locales. Participant email addresses and IP addresses were stored and audited to prevent duplicates, and as an internal quality control measure. An international collaborative authorship model was chosen to expand global reach of the survey. Collaborative studies have expanded global research networks and increased participation in research [15].

### Independent variables

This survey collected data on 14 independent variables. These included participant demographic data such as age, gender and country of residence; their current stage of their medical student training (pre-clinical or clinical); a diagnosis of symptomatic Covid-19 infection; increase in clinical responsibility; changes to teaching methods (non-virtual: lectures, tutorials, ward-based teaching sessions, theatre sessions, conferences and simulation sessions; virtual: online lectures and online tutorials, webinars or conferences).

### Participant experiences and outcomes

Data were recorded on participant experiences during the pandemic. Participants reported changes to placement duration and whether their examinations were postponed. The impact on their preparation for the next stage of training, learning opportunities, confidence in clinical and procedural skills, and choice of future career speciality were also surveyed. Participants also reported on the level of supervision received when performing clinical tasks during this period such as clerking and admitting patients, performing clinical procedures, and independently assessing or managing acutely unwell patients. Finally, participants were asked to rate the overall perceived impact on their undergraduate medical training using a Likert scale.

### Data analysis

Data were collated using Excel (Microsoft, Redmond, Washington, USA). Non-normal data were presented as median and interquartile range (IQR). Categorical data were summarised as proportions and percentages in tables. Country of residence was categorised low, middle or high income based on data from the World Bank [16]. Participant responses acquired in the form of Likert scales or a categorical range were combined to generate binary data. Statistical analysis was performed using SPSS (IBM, New York, USA). Univariate (unadjusted) analyses were performed using χ2-tests to assess the statistical significance in associations between 14 independent variables (Table 1 and Table 2) and participant reported overall negative impact on undergraduate medical training. The same method was used to assess the significance in associations between 9 participant experiences (Table 3) and country of residence and stage of undergraduate training. Multivariable (adjusted) analysis was performed between the former 14 independent variables and participant reported overall impact on undergraduate medical training. The results from the binary logistic regression analysis were displayed as odds ratios (OR) and 95% confidence intervals. Multicollinearity of the 14 independent variables was assessed by calculating their variance inflation factors (VIFs). A *p*-value of < 0.05 was assigned as the level of statistical significance.

**Table 1:** Factors associated with student reported overall negative impact on medical student training during the Covid-19 pandemic

	Total	Reported an overall negative impact on medical student training		
	%	Yes (%)	No (%)	***p-***value^*^
Total	1604 (100%)	1305 (81.4%)	299 (18.6%)	..
Age (years)^a^				
*≤ 21*	842 (100%)	715 (84.9%)	127 (15.1%)	**0.001**
*> 21*	762 (100%)	590 (77.4%)	172 (22.6%)	
Gender				
*Female*	860 (100%)	722 (84.0%)	138 (16.0%)	0.004**
*Male*	725 (100%)	570 (78.6%)	155 (21.4%)	
*Prefer not to say*	19 (100%)	13 (68.4%)	6 (31.6%)	
Stage of undergraduate training				
*Clinical*	796 (100%)	636 (79.9%)	160 (20.1%)	0.136
*Pre-clinical*	808 (100%)	669 (82.8%)	139 (17.2%)	
Resident nation economy				
*Low/middle income*	1356 (100%)	1104 (81.4%)	252 (18.6%)	0.891
*High income*	248 (100%)	201 (81%)	47 (19%)	
Contracting symptomatic Covid-19 infection				
*Yes*^c^	201 (100%)	152 (75.6%)	49 (24.4%)	**0.026**
*No*	1403 (100%)	1153 (82.2%)	250 (17.8%)	
Increased clinical responsibilities				
*Reported increase*	430 (100%)	314 (73%)	116 (27%)	**< 0.001**
*Did not report increase*^d^	1174 (100%)	991 (84.4%)	183 (15.6%)	

^*^Pearson χ2 statistical test used for univariate analysis to obtain *p*-values

**Combined analysis between female vs. male and prefer not to say groups

^a^ Twenty one chosen as age category cut-off as this was the median age

^b^Includes all with symptoms and diagnosed on a PCR swab test, antibody test, or by a clinician or self-diagnosed based on symptoms as per the World Health Organisation criteria

^c^ Includes all who stated not applicable

**Table 2:** Changes in teaching methods during the pandemic and association with student reported overall negative impact on medical student training

	Total	Reported an overall negative impact on medical student training		
	%	Yes (%)	No (%)	***p-***value^***+***^
Total	1604 (100%)	1305 (81.4%)	299 (18.6%)	..
Non-virtual methods				
Lectures				
*Declined*	941 (100%)	814 (86.5%)	127 (13.5%)	**< 0.001**
Did not report decline*	663 (100%)	491 (74.1%)	172 (25.9%)	
Tutorials				
*Declined*	808 (100%)	691 (85.5%)	117 (14.5%)	**< 0.001**
Did not report decline*	796 (100%)	614 (77.1%)	182 (22.9%)	
Ward based teaching session				
*Declined*	967 (100%)	825 (85.3%)	142 (14.7%)	**< 0.001**
Did not report decline*	637 (100%)	480 (75.4%)	157 (24.6%)	
Theatre sessions				
*Declined*	826 (100%)	697 (84.4%)	129 (15.6%)	**0.001**
Did not report decline*	778 (100%)	608 (78.1%)	170 (21.9%)	
Conferences				
*Declined*	626 (100%)	532 (85%)	94 (15%)	**0.003**
Did not report decline*	978 (100%)	773 (79%)	205 (21%)	
Simulation sessions				
*Declined*	574 (100%)	498 (86.8%)	76 (13.2%)	**< 0.001**
Did not report decline*	1030 (100%)	807 (78.3%)	223 (21.7%)	
Virtual methods				
Online lectures				
*Increased*	1476 (100%)	1191 (80.7%)	285 (19.3%)	**0.020**
Did not report increase**	128 (100%)	114 (89.1%)	14 (10.9%)	
Online tutorials, webinars or conferences				
*Increased*	1369 (100%)	1101 (80.4%)	268 (19.6%)	**0.020**
Did not report increase**	235 (100%)	204 (86.8%)	31 (13.2%)	

+Pearson χ2 statistical test used for univariate analysis to obtain p-values

* Includes all participants who reported increased, significantly increased, no change and not applicable. ** Includes all participants who reported decreased, significant decreased, no change and not applicable

**Table 3:** Undergraduate medical student experiences during the pandemic by resident nation economy and stage of training

	Resident of low or middle income country			Stage of undergraduate training		
	Yes (%)	No (%)	***p-***value^*^	Clinical (%)	Pre-Clinical (%)	***p-***value^*^
Total	1356 (100%)	248 (100%)	..	796 (100%)	808 (100%)	..
Placement duration						
*Reported shortened duration*	630 (46.5%)	152 (61.3%)	**< 0.001**	432 (54.3%)	350 (43.3%)	**< 0.001**
*Did not report a shortened duration*	726 (53.5%)	96 (38.7%)		364 (45.7%)	458 (56.7%)	
Examinations						
*Reported postponement*	1016 (74.9%)	132 (53.2%)	**< 0.001**	567 (71.2%)	581 (71.9%)	0.765
*Did not report postponement*	340 (25.1%)	116 (46.8%)		229 (28.8%)	227 (28.1%)	
Preparation for the next stage in training						
*Reported preparation was affected*	1054 (77.7%)	220 (88.7%)	**< 0.001**	663 (83.3%)	611 (75.6%)	**< 0.001**
*Did not report being affected*	302 (22.3%)	28 (11.3%)		133 (16.7%)	197 (24.4%)	
Learning opportunities						
*Reported a reduction*	1126 (83%)	211 (85.1%)	0.427	672 (84.4%)	665 (82.3%)	0.254
*Did not report a reduction*	230 (17%)	37 (14.9%)		124 (15.6%)	143 (17.7%)	
Confidence in clinical skills						
*Reported confidence was affected*	989 (72.9%)	210 (84.7%)	**< 0.001**	670 (84.2%)	529 (65.5%)	**< 0.001**
*Did not report being affected*	367 (27.1%)	38 (15.3%)		126 (15.8%)	279 (34.5%)	
Choice of future speciality						
*Reported being affected*	813 (60%)	130 (52.4%)	**0.027**	508 (63.8%)	435 (53.8%)	**< 0.001**
*Did not report being affected*	543 (40%)	118 (47.6%)		288 (36.2%)	373 (46.2%)	
Clerking-in patients without adequate supervision						
*Reported*	103 (7.6%)	26 (10.5%)	0.153	91 (11.4%)	38 (4.7%)	**< 0.001**
*Did not report*	1253 (92.4%)	222 (89.5%)		705 (88.6%)	770 (95.3%)	
Performing clinical procedures without adequate supervision						
*Reported*	133 (9.8%)	21 (8.5%)	0.509	114 (14.3%)	40 (5%)	**< 0.001**
*Did not report*	1223 (90.2%)	227 (91.5%)		682 (85.7%)	768 (95%)	
Assessing or managing acutely unwell patients independently without adequate supervision						
*Reported*	154 (11.4%)	21 (8.5%)	0.180	120 (15.1%)	55 (6.8%)	**< 0.001**
*Did not report*	1202 (88.6%)	227 (91.5%)		676 (84.9%)	753 (93.2%)	

^*^Pearson χ2 statistical test used for univariate analysis to obtain p-values

## Results

A total of 1625 participants took part in the survey of whom 1604 met the eligibility criteria. The median age was 21 (IQR: 21–23). Female students accounted for 53.6% (*n* = 860) of participants whilst 45.2% (*n* = 725) were male and 1.2% (*n* = 19) preferred not to disclose their gender (Table 1). Just under a half (*n* = 796, 49.6%) of medical students were in clinical years and the remainder (50.4%, *n* = 808) were in pre-clinical years. The majority (*n* = 1356, 84.5%) were residents of a low or middle income country. A full list of participant countries of residence can be found in Supplementary Table 1. Approximately 12.5% (*n* = 201) reported contracting symptomatic Covid-19 infection. Approximately a quarter (*n* = 430, 26.8%) of students reported an increase in clinical responsibilities during the pandemic.

Fifty to 60 % of students reported a decline in face-to-face lectures (58.6%, *n* = 941), tutorials (n = 808, 50.4%), ward-based teaching sessions (*n* = 967, 60.3%) and theatre sessions (*n* = 826, 51.5%) (Table 2). Over a third reported a decline in conferences (*n* = 626, 39.0%) and simulation sessions (*n* = 574, 35.7%). Around 9 out of 10 students reported an increase in the use of virtual learning methods such as online lectures (*n* = 1476, 92.0%) and online tutorials / webinars / conferences (*n* = 1369, 85.3%). Approximately half (*n* = 782, 48.7%) reported that their placement length was shortened and 71.6% (*n* = 1148) reported that their examinations were postponed.

Nearly 8 out of 10 students reported that the preparation for the next stage of their training (*n* = 1274, 79.4%) and their learning opportunities (*n* = 1337, 83.3%) were affected due to the pandemic (Table 3). Whilst 74.7% (*n* = 1199) reported their confidence in performing clinical skills was affected, only 58.8% (*n* = 943) reported that the pandemic had any impact on their future career specialty of choice. A minority reported performing clinical tasks without adequate supervision during the pandemic, with 8.0% (*n* = 129) reporting having to clerk-in patients without adequate supervision; 9.6% (*n* = 154) reporting having to perform clinical procedures without adequate supervision and 10.9% (*n* = 175) reporting having to independently assess or manage acutely unwell patients.

### Factors associated with an overall negative impact on medical student training

Overall, 81.4% (*n* = 1305) participants reported that the Covid-19 pandemic had a negative impact on their medical student training, whilst 18.6% (*n* = 299) did not report a negative impact. On univariate analysis female gender, a decline in face-to-face lectures, tutorials, ward-based teaching, theatre sessions, conferences and simulation sessions were associated with significantly higher proportion of students reporting an overall negative impact on training compared to the participants who were not female or did not report on a decline in any of the former teaching methods (*p <* 0.05; Table 1 and Table 2). In contrast, students who contracted symptomatic Covid-19 infection, those who reported an increase in clinical responsibilities and those who observed an increase in online lectures, tutorials, webinars or conferences, all reported an overall negative impact on their training (*p <* 0.05; Table 1 and Table 2).

Covariate adjusted binary logistic regression analysis was performed for 1604 participants and 14 independent variables (Table 1 and Table 2) comparing participant reported overall negative impact on training, as the outcome variable. Accounting for confounders, being aged 21 or younger or female were associated with 60 and 40% greater odds respectively of reporting an overall negative impact on their training (*p* = 0.004 and *p* = 0.007 respectively, Table 4). An increase in clinical responsibility was associated with 40% less odds of medical students reporting a negative impact on training (*p* < 0.001). However, a decline in face-to-face lectures and ward-based teaching were associated with 90 and 60% greater odds of students reporting a negative impact on their training following the Covid-19 pandemic (*p ≤* 0.001), respectively. Multicollinearity assessment of the 14 independent variables revealed that all VIFs were less than 2 (maximum 1.5), indicating the validity of including all these independent variables in the logistic regression model (Supplementary Table 2).

**Table 4:** Adjusted analysis of factors associated with medical students reporting an overall negative impact on training during the Covid-19 pandemic

	Reported an overall negative impact on medical student training
*Age ≤ 21*	**1.6 (1.2–2.1);** ***p =*** **0.004**
*Female gender*	**1.4 (1.1–1.9);** ***p =*** **0.007**
*Clinical years*	1.1 (0.8–1.5); *p = 0.700*
*Low or middle income country resident*	1.2 (0.8–1.7); *p* = 0.362
*Contracted Covid-19 infection*	0.7 (0.5–1.1); *p* = 0.114
*Increased clinical responsibility*	**0.6 (0.4–0.8); p < 0.001**
*Decline in lectures (non-virtual)*	**1.9 (1.4–2.5);** ***p <*** **0.001**
*Decline in tutorials (non-virtual)*	1.1 (0.8–1.4); *p =* 0.721
*Decline in ward based teaching (non-virtual)*	**1.6 (1.2–2.2);** ***p*** **= 0.001**
*Decline in theatres sessions (non-virtual)*	1.0 (0.8–1.4); *p* = 0.881
*Decline in conferences (non-virtual)*	1.2 (0.9–1.6); *p = 0.299*
*Decline in simulations (non-virtual)*	1.3 (0.9–1.8); *p =* 0.103
*Increase in virtual / online lectures*	0.8 (0.4–1.5); *p = 0.490*
*Increase in virtual / online tutorials, webinars and conferences*	0.9 (0.6–1.3); *p = 0.482*

Binary logistic regression analysis was performed with the 14 independent variables listed in this table. Significant results have been highlighted in bold

### Medical student training experiences during the pandemic

Univariate analysis also demonstrated that a significantly lower proportion of medical students who were residents of low or middle income countries reported that their placement duration was shortened, their preparation for the next stage in training and confidence in clinical skills were affected due to the pandemic (*p* < 0.001; Table 3). In contrast, a significantly lower proportion of students residing in higher income countries reported that their examinations were postponed, and their choice of future career speciality was affected due to the pandemic (*p* < 0.05). A significantly higher proportion of medical students in the clinical years of their studies reported that their placements were shortened, their preparation for the next stage of training, confidence in clinical skills and choice of future career speciality were affected (*p* < 0.001). Whilst the proportions of students performing clinical tasks without adequate supervision was low across clinical and pre-clinical years, a significantly higher proportion in clinical years reported having to clerk-in patients, perform clinical procedures and assess or manage acutely unwell patients during the pandemic (*p* < 0.001).

## Discussion

The findings from this survey revealed that most medical students perceived that the Covid-19 pandemic had an overall negative impact on their training (81.4% vs. 16.4%). Allowing for confounders, younger students were 60% more likely to report a negative impact on training. Pre-clinical medical students rely heavily on active teaching methods [17]. Poor pedagogy has been associated with negative learning experiences amongst early year medical students [18]. The pandemic and subsequent prolonged periods of lockdown led to a substantial reduction in the volume of teaching received by medical students. This likely had a greater impact on younger students in pre-clinical years, who rely heavily on pedagogical methods compared to their older counterparts who rely on andragogy. Furthermore, students reporting a reduction in conventional lectures and ward-based teaching were also significantly more likely report a negative impact on training. In contrast, an increase in clinical responsibilities was less likely to be associated with medical students reporting a negative impact on training. However, being a student in clinical years was not associated with participants reporting a negative impact.

Medical students reported a large increase in novel virtual teaching methods such as online lectures, tutorials, webinars and conferences during the pandemic. However, increased use of these methods was not significantly associated with medical students reporting an overall negative impact on training. Several authors have previously raised concerns regarding the efficacy of such novel virtual teaching methods in medical education [14, 19]. Limited resources, poor infrastructure and technical difficulties are significant barriers to virtual medical training [20, 21]. Further research comparing these novel methods with traditional methods using validated measurable outcomes is needed to objectively assess their efficacy. A decline in theatre sessions, conferences or simulation-based teaching were not significantly associated with participants reporting a negative impact on training. This might be due to most of the participants surveyed being students registered in pre-clinical years. Theatre sessions and simulation are less commonly used teaching methods amongst pre-clinical students. The survey also yielded several unexpected findings. A higher proportion of female participants reported a negative impact on training. The reasons behind this observation are unclear. Furthermore, contracting Covid-19 infection was not significantly associated with a negative impact on training. Further research is needed to explore the reasons behind these observations.

The results demonstrated that certain participant experiences during the pandemic were associated with being a student in clinical years of training or being a resident of a low or middle income country. Many of these could be attributed to technical challenges faced by students as well as faculty in low or middle income countries when implementing virtual teaching methods due to limited resources being available [19]. A recent survey of 3348 medical students from Libya found that around two thirds (64.7%) felt that e-learning could not be easily implemented in their country [22]. Whilst only a very small proportion of students reported having to perform any form of clinical tasks without adequate supervision, this was significantly more frequently reported by students in clinical years. This is unsurprising, given the limited clinical exposure during pre-clinical years of medical training. There was no significant difference in the proportions of students reporting having to perform clinical tasks without adequate supervision and the participant’s resident nation economic status. Medical students reported that they were expected to perform clinical tasks without adequate supervision during the pandemic. The latter could lead to detrimental effects on student wellbeing [23]. Whilst greater autonomy and reduced supervision amongst senior trainee doctors could increase their confidence, there is no evidence to support the notion that this could be extrapolated to medical students [24]. It is important to note that these significant associations identified between the nine participant experiences and medical student level of training or participant’s resident nation economic status (Table-3) were based on univariate analyses. A multivariable analysis taking into consideration any confounding factors is required to determine statistically reliable associations.

There were several limitations in this study. The use of a cross sectional survey is inherently at risk of responder bias. The sample size was relatively small compared to the large target demographic chosen. The study was only conducted in the English language. There was no in-depth evaluation of medical student social circumstances or wellbeing. The main outcome measure was not a validated objective measure. Therefore, these finding must be interpreted with caution. Given such limitations, future research evaluating the impact of the pandemic on medical student training and education or the impact of novel virtual teaching methods on medical students, should focus on specific interventions, target populations and adopt quantitative outcome measures (e.g., standardised exam grades). There were also several strengths. This is one of the first international surveys conducted through a global collaborative effort to evaluate the impact of the Covid-19 pandemic on medical student training. It is one of the first to objectively assess the changes in methods of medical student teaching. It had one of the largest sample sizes from the published literature pertaining to this topic at the time of publication.

## Conclusion

The Covid-19 pandemic led to negative experiences associated with training for many medical students. This survey found that medical students who were younger, female, who did not receive conventional face-to-face lectures or ward-based teaching were more likely to report a negative impact on training. Having greater clinical responsibility offset student perceived negative experiences in training during the pandemic. We have shown that medical students training was affected by the pandemic and any lasting impact is yet to be determined. As medical schools and teaching hospitals emerge from the pandemic it is necessary to allocate additional resources to cater to the students whose training affected during the pandemic. Whilst there has been a proliferation in the use of novel virtual teaching methods globally, more research is needed to explore the efficacy of these novel teaching tools.

## Supplementary Information

**Additional file 1:.**

**Additional file 2:.**

**Additional file 3:.**

**Additional file 4:.**

**Additional file 5:.**

## Data Availability

All relevant data and results included in this article have been published along with the article and its supplementary information files. Anonymised data can be obtained on reasonable request from the corresponding author.

## References

[1] Ahmed H, Allaf M, Elghazaly H (2020). COVID-19 and medical education. Lancet Infect Dis.

[2] Samaraee AA. The impact of the COVID-19 pandemic on medical education. In: MA Healthcare London. 2020. 10.12968/hmed.2020.0191 32730144

[3] Choi B, Jegatheeswaran L, Minocha A, Alhilani M, Nakhoul M, Mutengesa E (2020). The impact of the COVID-19 pandemic on final year medical students in the United Kingdom: a national survey. BMC Medical Education.

[4] Akers A, Blough C, Iyer MS (2020). COVID-19 implications on clinical clerkships and the residency application process for medical students. Cureus.

[5] Komer L (2020). COVID-19 amongst the pandemic of medical student mental health. International Journal of Medical Students.

[6] Sahu P (2020). Closure of universities due to coronavirus disease 2019 (COVID-19): impact on education and mental health of students and academic staff. Cureus.

[7] Meo SA, Abukhalaf AA, Alomar AA, Sattar K, Klonoff DC: COVID-19 pandemic: impact of quarantine on medical students’ mental wellbeing and learning behaviors. Pakistan journal of medical sciences 2020, 36(COVID19-S4):S43. 10.12669/pjms.36.COVID19-S4.2809 PMC7306952 32582313

[8] Compton S, Sarraf-Yazdi S, Rustandy F, Radha Krishna LK (2020). Medical students’ preference for returning to the clinical setting during the COVID-19 pandemic. Med Educ.

[9] Sandhu P, de Wolf M (2020). The impact of COVID-19 on the undergraduate medical curriculum. Medical Education Online.

[10] Abi-Rafeh J, Azzi AJ: Emerging role of online virtual teaching resources for medical student education in plastic surgery: COVID-19 pandemic and beyond. Journal of Plastic, Reconstructive & Aesthetic Surgery 2020. 10.1016/j.bjps.2020.05.085 PMC7256619 32553546

[11] Liang ZC, Ooi SBS, Wang W. Pandemics and their impact on medical training: lessons from Singapore. Acad Med. 2020. 10.1097/ACM.0000000000003441 PMC7188065 32304387

[12] Hickland MM, Gosney ER, Hare KL: Medical student views on returning to clinical placement after months of online learning as a result of the COVID-19 pandemic. Medical Education Online 2020, 25(1). 10.1080/10872981.2020.1800981 PMC7482891 32723165

[13] Shahrvini B, Baxter SL, Coffey CS, MacDonald BV, Lander L (2021). Pre-clinical remote undergraduate medical education during the COVID-19 pandemic: a survey study. BMC Medical Education.

[14] Dost S, Hossain A, Shehab M, Abdelwahed A, Al-Nusair L (2020). Perceptions of medical students towards online teaching during the COVID-19 pandemic: a national cross-sectional survey of 2721 UK medical students. BMJ Open.

[15] Davidson K, Bell A, Riedy R, Sandoval C, Wegemer C, Clark T, Farrell C, Fishman B (2020). Russell J.

[16] Country and Lending Groups [https://datahelpdesk.worldbank.org/knowledgebase/articles/906519-world-bank-country-and-lending-groups].

[17] Waliany S, Caceres W, Merrell SB, Thadaney S, Johnstone N, Osterberg L (2019). Preclinical curriculum of prospective case-based teaching with faculty-and student-blinded approach. BMC medical education.

[18] Schei E, Johnsrud RE, Mildestvedt T, Pedersen R, Hjörleifsson S (2018). Trustingly bewildered. How first-year medical students make sense of their learning experience in a traditional, preclinical curriculum. Medical education online.

[19] O’Doherty D, Dromey M, Lougheed J, Hannigan A, Last J, McGrath D (2018). Barriers and solutions to online learning in medical education–an integrative review. BMC Medical Education.

[20] Lakbala P (2016). Barriers in implementing E-learning in Hormozgan University of Medical Sciences. Global J Health Sci.

[21] Attardi SM, Rogers KA (2015). Design and implementation of an online systemic human anatomy course with laboratory. Anat Sci Educ.

[22] Alsoufi A, Alsuyihili A, Msherghi A, Elhadi A, Atiyah H, Ashini A, Ashwieb A, Ghula M, Ben Hasan H, Abudabuos S (2020). Impact of the COVID-19 pandemic on medical education: medical students’ knowledge, attitudes, and practices regarding electronic learning. PLoS One.

[23] Greenhill J, Fielke KR, Richards JN, Walker LJ, Walters LK (2015). Towards an understanding of medical student resilience in longitudinal integrated clerkships. BMC Medical Education.

[24] Singman EL, Srikumaran D, Green L, Tian J, McDonnell P (2017). Supervision and autonomy of ophthalmology residents in the outpatient Clinic in the United States: a survey of ACGME-accredited programs. BMC medical education.

