# Supplementary material for: The perceived impact of the Covid-19 pandemic on medical student education and training – an international survey
Source: BMC Med Educ. 2021 Nov 9;21:566. doi: 10.1186/s12909-021-02983-3 (PMC8576461; doi:10.1186/s12909-021-02983-3)
Supplement: Supplementary file 2 — Additional file 2:. [file 12909_2021_2983_MOESM2_ESM.docx]

**Supplementary Document 2: Medical Student Survey**

**Section 1: Participant Demographics**

1. What is your email address?
2. What is your age?
3. What is your gender?
4. In what country do you currently work, train or study?
5. Which one of the following best describes your occupational status?

*(Please note that the answer you provide for this question will determine the rest of the questions in this survey. Therefore, choose the correct option for you)*

1. Have you had COVID-19?

*(Please select the most appropriate answer for you.
TIP: Here, COVID-19 refers to the illness caused by SARS Novel Coronavirus - 2 characterised by fever, cough, loss or smell, shortness of breath, etc. I
f your tests came back positive but you did not have any symptoms please select "No I did not have any symptoms of Covid-19")*

**Section 2: Impact of the COVID-19 pandemic on medical student teaching and learning**

1. What stage of your undergraduate medical training are you at?
2. How has your placement module or semester been affected by COVID-19

*(for each of the following please select Yes or No)*

1. Had to take on more clinical responsibility than before as a result of COVID-19
2. Reduced in length of time
3. Reduced learning opportunities available for myself
4. Examinations were postponed
5. To what extent has the frequency of the following methods of teaching or training you receive changed due to COVID-19

*(Please select a single option from significantly increased to significantly decreased or not applicable for each one of the statements in this question)*

1. In relation to the following, how often were you expected to carry out a clinical task or procedure without adequate supervision during the Covid-19 pandemic?

   *(Please select a single option from very frequently to very infrequently or not applicable for each one of the statements in question 10. If you were already skilled and competent at a particular skill below or had adequate supervision when performing this task, please select not applicable)*
2. Clerk in or admit patients
3. Performing clinical skills (such as cannulas or catheters)
4. Assessing and managing unwell patients

Please enter any additional comments where you felt unsupported or had to complete tasks beyond your competence without adequate supervision than normal:

1. To what extent have the following aspects of your training progression been negatively/ adversely affected due to COVID-19?

*Please select a single option from: a great deal to not at all or not applicable for each one of these statements:*

1. Choice of future career specialty
2. Sufficient preparation for next placement, module or semester
3. Confidence in clinical skills
4. Increased interest in medical education
5. Overall, how do you feel that the pandemic has impacted your undergraduate medical training this year?

*(please indicate on this line)*

No impact

Positive impact

Negative impact

**End of Survey – Thank you very much for taking the time to complete this survey.**
