# Supplementary material for: The perceived impact of the Covid-19 pandemic on medical student education and training – an international survey
Source: BMC Med Educ. 2021 Nov 9;21:566. doi: 10.1186/s12909-021-02983-3 (PMC8576461; doi:10.1186/s12909-021-02983-3)
Supplement: Supplementary file 3 — Additional file 3:. [file 12909_2021_2983_MOESM3_ESM.docx]

| Country | N | % |
| --- | --- | --- |
| *India* | 710 | 44.3% |
| *Pakistan* | 189 | 11.8% |
| *United Kingdom* | 140 | 8.7% |
| *Egypt* | 128 | 8.0% |
| *Jordan* | 96 | 6.0% |
| *Libya* | 69 | 4.3% |
| *United Arab Emirates* | 52 | 3.2% |
| *Mexico* | 37 | 2.3% |
| *Bulgaria* | 26 | 1.6% |
| *Nigeria* | 19 | 1.2% |
| *Greece* | 18 | 1.1% |
| *Bangladesh* | 15 | 0.9% |
| *Turkey* | 11 | 0.7% |
| *Yemen* | 10 | 0.6% |
| *Sudan* | 9 | 0.6% |
| *Malaysia* | 7 | 0.4% |
| *Saudi Arabia* | 7 | 0.4% |
| *Afghanistan* | 5 | 0.3% |
| *Ukraine* | 5 | 0.3% |
| *United States of America* | 5 | 0.3% |
| *Canada* | 4 | 0.2% |
| *Ghana* | 4 | 0.2% |
| *Ireland* | 4 | 0.2% |
| *Algeria* | 3 | 0.2% |
| *Czech Republic* | 3 | 0.2% |
| *Israel* | 3 | 0.2% |
| *State of Palestine* | 3 | 0.2% |
| *Syrian Arab Republic* | 3 | 0.2% |
| *Georgia* | 2 | 0.1% |
| *Italy* | 2 | 0.1% |
| *Australia* | 1 | 0.1% |
| *Bahamas* | 1 | 0.1% |
| *Bahrain* | 1 | 0.1% |
| *Belarus* | 1 | 0.1% |
| *Ethiopia* | 1 | 0.1% |
| *Germany* | 1 | 0.1% |
| *Grenada* | 1 | 0.1% |
| *Hungary* | 1 | 0.1% |
| *Iraq* | 1 | 0.1% |
| *Kenya* | 1 | 0.1% |
| *Malawi* | 1 | 0.1% |
| *Norway* | 1 | 0.1% |
| *Poland* | 1 | 0.1% |
| *Serbia* | 1 | 0.1% |
| *Sweden* | 1 | 0.1% |
| Total | 1604 | 100% |

Supplementary Table 1: List of participants by country of residence
