# Supplementary material for: The perceived impact of the Covid-19 pandemic on medical student education and training – an international survey
Source: BMC Med Educ. 2021 Nov 9;21:566. doi: 10.1186/s12909-021-02983-3 (PMC8576461; doi:10.1186/s12909-021-02983-3)
Supplement: Supplementary file 4 — Additional file 4:. [file 12909_2021_2983_MOESM4_ESM.doc]

| Independent variable | Variance inflation factors (VIF) |
| --- | --- |
| 10_AGE_MEDIAN | 1.402 |
| 10_FEMALE_GENDER | 1.024 |
| 10_LOW_MID_Country | 1.049 |
| 10_COVID_STATUS_YES | 1.027 |
| 10_CLINICAL_YEARS | 1.461 |
| 10_INCREASED_RESPONSIBILITY | 1.048 |
| 10_DECREASED_F2FTUTORIALS | 1.392 |
| 10_DECREASED_WARD_BASED | 1.266 |
| 10_DECR_EASEDTHEATRE | 1.400 |
| 10_DECREASED_SIMULATIONS | 1.259 |
| 10_DECREASED_F2F_LECTURES | 1.198 |
| 10_INCREASED_WEB_LECTURES | 1.167 |
| 10_INCREASED_WEBINARS | 1.172 |
| 10_DECREASED_CONFERENCES | 1.154 |

Supplementary Table 2: Variance Inflation Factors
