# Supplementary material for: The perceived impact of the Covid-19 pandemic on medical student education and training – an international survey
Source: BMC Med Educ. 2021 Nov 9;21:566. doi: 10.1186/s12909-021-02983-3 (PMC8576461; doi:10.1186/s12909-021-02983-3)
Supplement: Supplementary file 5 — Additional file 5:. [file 12909_2021_2983_MOESM5_ESM.docx]

**Appendix A: Author List**

**Corporate authorship title: The TMS Collaborative**

**The Pubmed citable author list - in order of publication**

**Writing committee (citable)**

Kasun Wanigasooriya (1) (2)

William Beedham (1) (2)

Ryan Laloo (1) (3)

Rama Santhosh Karri (1) (4)

**Steering committee (citable)**

Adnan Darr (1) (3)

Georgia R. Layton (1) (5)

Peter Logan (1) (6)

Yanyu Tan (1) (7)

Devender Mittapalli (1) (8)

Tapan Patel (9)

**Collaborative authors (citable)**

Vivaswan Dutt Mishra (10)

Osama Odeh (11)

Swathi Prakash (12)

Salma Elnomany (13)

Sri Ramya Peddinti (14)

Elorm Adzoa Daketsey (15)

Shardool Gadgil (16)

Ahmad Elmabri Mohammad Bouhuwaish (17)

Ahmad Ozair (18)

Sanchit Bansal (19)

Muhammed Elhadi (20)

Aditya Amit Godbole (21)

Ariana Axiaq (22)

Faateh Ahmad Rauf (23)

Ashna Ashpak (24)

(1) The Master Surgeon Trust, Worcestershire, United Kingdom

(2) College of Medical and Dental Science, University of Birmingham, United Kingdom

(3) Leeds Vascular Institute, Leeds General Infirmary, Leeds, United Kingdom.

(4) The Royal Wolverhampton NHS Trust, Wolverhampton, United Kingdom

(5) Department of Cardiac Surgery, University Hospitals of Leicester NHS Trust. United Kingdom

(6) Walsall Healthcare NHS Trust, Walsall. United Kingdom

(7) North East Deanery, United Kingdom

(8) University Hospitals Plymouth NHS Trust, Plymouth, United Kingdom

(9) Baroda Medical College, India

(10) Motilal Nehru Medical College, Allahabad, India

(11) University of Jordan, Amman, Jordan

(12) HCG Cancer Centre, Bangalore, India

(13) Faculty of Medicine, Menoufia University, Menoufia, Egypt

(14) Indira Gandhi Medical College and Research Institute , Puducherry, India

(15) Ysbyty Gwynedd, Betsi Cadwaladr University Health Board, Gwynedd, Bangor, North Wales

(16) Lokmanya Tilak municipal medical college, Mumbai, India

(17) Faculty of Medicine Tobruk University, Tobruk, Libya

(18) Faculty of Medicine, King George's Medical University, Lucknow, Uttar Pradesh, India.

(19) Vardhman Mahavir Medical College and Safdarjung Hospital, New Delhi, India

(20) Faculty of Medicine, University of Tripoli, Tripoli, Libya

(21) Bharati Vidyapeeth (Deemed to be University) Medical College, Dhankawadi, Pune, India.

(22) School of Medicine, Faculty of Life Sciences, Queen's University Belfast, Belfast, UK

(23) Combined Military Hospital Lahore Medical College, Lahore, Pakistan

(24) School of Medicine, University of Central Lancashire, Preston, Lancashire, United Kingdom.
